# Cross-Cultural Color-Odor Associations

**DOI:** 10.1371/journal.pone.0101651

**Published:** 2014-07-09

**Authors:** Carmel A. Levitan, Jiana Ren, Andy T. Woods, Sanne Boesveldt, Jason S. Chan, Kirsten J. McKenzie, Michael Dodson, Jai A. Levin, Christine X. R. Leong, Jasper J. F. van den Bosch

**Affiliations:** 1 Department of Cognitive Science, Occidental College, Los Angeles, California, United States of America; 2 Division of Human Nutrition, Wageningen University, Wageningen, the Netherlands; 3 Xperiment, Lausanne, Switzerland; 4 Institute for Medical Psychology, Goethe University, Frankfurt am Main, Germany; 5 School of Psychology, The University of Nottingham Malaysia Campus, Semenyih, Selangor Darul Ehsan, Malaysia; 6 Institute for Learning and Brain Sciences, University of Washington, Seattle, Washington, United States of America; Technical University of Dresden Medical School, Germany

## Abstract

Colors and odors are associated; for instance, people typically match the smell of strawberries to the color pink or red. These associations are forms of crossmodal correspondences. Recently, there has been discussion about the extent to which these correspondences arise for structural reasons (i.e., an inherent mapping between color and odor), statistical reasons (i.e., covariance in experience), and/or semantically-mediated reasons (i.e., stemming from language). The present study probed this question by testing color-odor correspondences in 6 different cultural groups (Dutch, Netherlands-residing-Chinese, German, Malay, Malaysian-Chinese, and US residents), using the same set of 14 odors and asking participants to make congruent and incongruent color choices for each odor. We found consistent patterns in color choices for each odor within each culture, showing that participants were making non-random color-odor matches. We used representational dissimilarity analysis to probe for variations in the patterns of color-odor associations across cultures; we found that US and German participants had the most similar patterns of associations, followed by German and Malay participants. The largest group differences were between Malay and Netherlands-resident Chinese participants and between Dutch and Malaysian-Chinese participants. We conclude that culture plays a role in color-odor crossmodal associations, which likely arise, at least in part, through experience.

## Introduction

Would a rose smell as sweet if it were blue? Perhaps not; color plays an important role in recognizing odors and congruent color-odor combinations are rated as more pleasant than incongruent combinations [1]. Colors and odors can be consistently matched across participants [2]–[5]; for instance, caramel tends to be most commonly associated with brown and strawberry with pink. Color influences odor identification, discrimination, intensity, and even pleasantness (see [6] for a thorough review). Thus, crossmodal correspondences between colors and odors exist.

Crossmodal correspondences can take different forms. Spence [7] distinguishes between three kinds of correspondences: structural, statistical, and semantically mediated. Structural correspondences can occur due to the interplay of neural correlates (in some cases due to various stimuli exciting different sensory organs sharing a feature such as magnitude or intensity). Statistical correspondences are learned, and occur when two stimulus dimensions are routinely correlated in the environment. Semantically mediated correspondences arise due to language, such as “low” being used to refer both to elevation and pitch. The type of crossmodal effect has implications for the perceptual consequences of the correspondence. Semantically-mediated correspondences are likely post-perceptual, but structural and statistical correspondences could potentially lead to perceptual or post-perceptual effects. For color-odor associations, both perceptual and semantic factors seem to play a role; color brightness correlates with perceptual attributes of odors (odors that are more irritating, intense, and unpleasant are associated with brighter colors) and semantic attributes (more familiar and identifiable odors are associated with more saturated colors), though the role of hedonics was also important [8]. Indeed, the congruency of color-odor pairs is reflected in the activity of brain areas associated with the hedonics of smell [9]. Spence's tripartite classification [7] thus provides a useful theoretical framework for considering the nature of crossmodal correspondences.

Cross-cultural comparisons allow some insight into how these correspondences might emerge. Structural correspondences might be expected to be either highly idiosyncratic (in the case of synesthesia) or universal (if they reflect an underlying neural mechanism common to all people). Some statistical correspondences are also likely to be universal (e.g. larger objects tend to have lower resonant frequencies) but others may be less universal if environments are likely to differ. Finally, semantically mediated correspondences are more context dependent; as language influences these associations, different cultures may experience different crossmodal associations.

Comparing different studies that used similar stimuli has the potential to provide some insight into this question. For example, potential evidence for structural correspondences (in the universal sense) between color and odor has come from cases in which colors are reliably associated with odors where there is not a likely history of learning, such as for almond [10]. In that study, which was conducted in Canada, almond was significantly associated with red (and also with purple and gray). If the authors' claim that the correspondence was not likely to have been learned is correct, then this result would support the notion of a structural correspondence. However, comparing cultures demonstrates that the red-almond association is not universal, as in an Australian sample, almond odor was associated with blue [8]. This difference would seem to imply that the correspondence could not be structural. But there was variation in the odors used (as well as their concentration) and the ways in which the color matches were obtained and analyzed. In the Canadian study [10], participants generated a verbal color label and were instructed not to try to identify the odor, while in the Australian study [8], participants made a match to a visual color after attempting to name the odor. Therefore, it is possible that procedural, rather than cultural, factors underlie the differences in results. Labels can change the perception of odors [11], and different intensities of the same odor can result in differences in color matches to that odor [5], [12]. Thus the difference between the Canadian and Australian color matches to almond does not rule out the existence of structural correspondences. Because of diversity in stimuli and experimental approach, comparison of different studies cannot fully address this question of universality; studies explicitly designed to examine cultural factors can rule out these procedural differences.

Cultural differences in odor perception have been identified. In the US, anise, wintergreen and cinnamon odors are associated with sweets; in France, they are considered medicinal; and in Vietnam they are classified as floral but associated with traditional medicine [13]. Culturally-specific emotional experiences with particular odors may explain differences in how pleasant the odors are perceived to be; wintergreen has been rated as very pleasant in the US, where it has been associated with candy but as very unpleasant in the UK, where it is associated with medicine [14]. Studies of these cultural differences in odor preferences have been systematically investigated since Pangborn, Guinard, and Davis studied 16 different groups and found that the patterns of odor preferences could be clustered into distinct groups (e.g. all 7 European countries in their sample clustered together) and that both country and ethnic origin influenced liking of particular odors; for instance, ethnic Taiwanese people living in California showed similar preferences to both non-Taiwanese Californians and to Taiwanese people living in Taiwan [15]. Subsequent studies have found that people in different cultures even rate the intensity of many odors differently [13], [16], [17]. As intensity evaluations do not require judgments of identity or pleasantness of the odors, these differences in perceived intensity are particularly noteworthy; while experience with odors at particular concentrations may differ (e.g., due to cultural uses of particular spices while cooking), evaluation of intensity is likely predominantly a perceptual task.

Culture's influence on color perception is more controversial. Color categories seem to be near universal, but there are some cultural differences in these categories; color categories in turn can influence perception, though the effect may be stronger in the right visual field than in the left (see [18] for a review). For instance, Russian speakers use different terms for light and dark blues, and are faster at distinguishing shades of blue that cross their linguistic boundary than distinguishing shades that do not, but English speakers are not faster at distinctions that cross the Russian boundary. When a spatial interference task is added, the cross-boundary advantage persists for Russian speakers but when a language interference task is added, the cross-boundary advantage disappears [19]. Thus the perceptual differences seem dependent on access to language as they are semantically mediated.

The present study aims to probe the nature of crossmodal correspondences by comparing color-odor associations in participants from different cultural backgrounds, using odors that occur across cultures and using a non-verbal task. If these associations are universal, they are unlikely to be semantically mediated, but if they differ systematically by group, then the crossmodal correspondence between color and odor cannot be structural. A previous cross-cultural study asked British and Taiwanese participants to look at pictures of colored drinks and state what flavor they would expect to experience; that study found systematic differences in expectations, such as brown drinks being associated with cola in the UK and grape in Taiwan [20]. A more recent study [21] presented participants in the US and in China with photographs of colored beverages in different receptacles and asked them to select the flavor (from a list) that first came to mind and also found cross-cultural differences. Both sets of results are likely due to different patterns in beverage consumption in those nations, which could lead to a statistical or a semantic correspondence. Our study uses actual odors and asks participants to select the most congruent and incongruent colors to systematically map out the pattern of association for each group.

Our goal is not just to find the single color most strongly associated with an odor, but instead to map color-odor associations across a wider palette of colors. Summary statistic approaches based on the univariate frequency of a chosen color ignore more subtle interactions with weaker associated colors, or opposing colors. Instead, a multivariate approach is employed to take into account groups of colors, such as bright colors, pastel colors, warm versus cold colors, without explicitly defining these categories.

We apply a representational similarity analysis [22] because we are principally interested in how a distribution of congruent and incongruent colors represents various odors, and the degree to which cultures are similar or differ in their patterns. This technique has proven successful in neuroimaging studies comparing physically distinct data, such as semantic categories in monkey and human object areas [23].

Our approach allows us to compare color-odor associations, both for specific odors and for the pattern of color-odor associations across cultures. Consistent associations within a culture but differences in patterns across cultures would demonstrate that color-odor associations are not simply structural, but that they are mediated by statistical or semantic experiences.

## Methods

### Ethics Statement

Participants at all sites gave written consent to participate in the study. The Germany and US protocols were both approved by the Occidental College Human Subjects Research Review Committee. The Malaysia protocol was approved by the University of Nottingham Malaysia Campus Research Ethics Committee. At the time of the study, Wageningen University (where the Dutch studies were carried out) did not require committee review of social science research (only of medical or animal research, and this study was not considered medical).

### Design

Six different populations of participants completed the same task of selecting colors that were the most and least consistent for each of 14 different odorants. Culture was a between-participants variable. Odor and consistency/inconsistency were the within-participant variables.

### Participants

A total of 122 untrained participants from six populations were recruited: Dutch, Netherlands-residing Chinese, German, Malay, Malaysian-Chinese, and US resident. Dutch (twenty participants, 9 female, mean age 24 years) and Netherlands-residing Chinese participants (20 participants, 9 female, mean age 26 years) were tested in the Netherlands. Twenty German participants were tested in Germany (13 female, mean age 28 years). Malay (20 participants, 16 female, mean age 21 years) and Malaysian-Chinese participants (20 participants, 15 female, mean age 20 years) were tested in Malaysia. Twenty-two participants were tested in the US (18 female, mean age 20 years). The Netherlands-residing Chinese participants were native Chinese people (born and raised in China) who had resided in the Netherlands for less than two years; all other participants had grown up in the country in which they were tested. In Malaysia, national identity cards specify race/ethnicity and so recruitment advertisements explicitly asked for participants who identified themselves as ‘Malay’ or ‘Malaysian Chinese’ and participants were asked to state their race as listed on their card; only participants who indicated ‘Malay’ or ‘Malaysian Chinese’ were included in the data analysis for this study. Because our hypothesis is that differential experiences between groups would lead to differences in color-odor associations, we viewed our categories as likely to reflect diversity in practices such as consumption of particular foods and use of other scented items.

All participants were healthy volunteers who reported a normal sense of smell and no history of olfactory impairments or current respiratory complications (e.g. colds or allergies). Participants received extra credit in coursework (in Malaysia and the US), cash remuneration (€10 in Germany; €5 in the Netherlands), or chocolate/fruit (in Malaysia) for their participation.

### Materials

**Figure 1:**
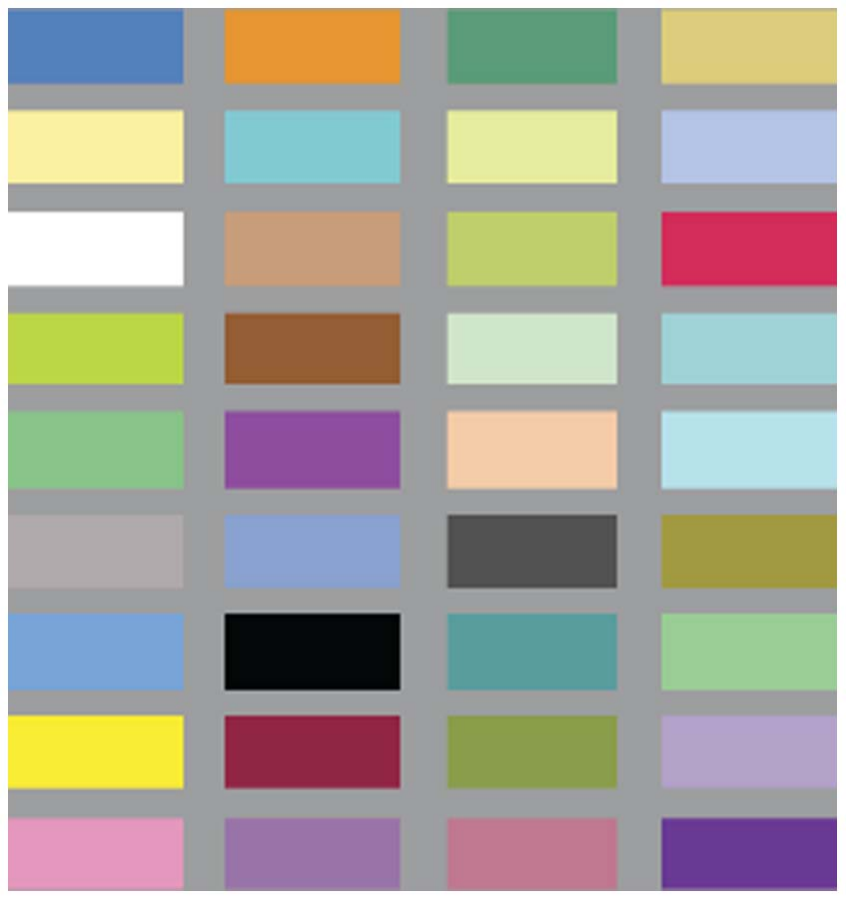
Color choices. 36 colors were arranged in random order for each trial.

Fourteen odorants were embedded in odor pens. Odor pens work in a similar fashion to felt tip pens, but instead of being loaded with ink-based color, they are filled with odorant instead. The odorants were originally used for industrial research and were designed to typify the following descriptions: burnt, candy, fish, flower, fruity, hazelnut, meat, musty, plastic, rice, soap, vegetable, vinegar, and woody. These specific odors were selected as they were likely to be common across cultures and were easily discriminable. Please contact the corresponding author for additional information about the chemical composition of the odorants. Thirty-six colors, derived from the 32 used in the Berkeley Color Project [24] with the addition of white, light gray, dark gray, and black were randomly arranged for each trial as in Figure 1. The visual stimuli were presented through the Xperiment software package (version 0.0.12; www.xperiment.mobi) either on an Android phone (HTC Desire Z, Android 2.2 (Froyo) with HTC Sense) in the Netherlands or on an iPod touch 3^rd^ Generation in Germany, Malaysia, and the US. A control experiment comparing the iPod and the Android displays found no significant difference in color associations using the different devices.

### Procedure

Participants were given each odor pen one at a time, in random order. Participants indicated the three most congruent and three most incongruent colors for each pen by selecting those colors on screen; no verbal labels for color or odor were given, as we did not wish to activate any semantic associations. We asked participants to select both congruent and incongruent colors as this approach had been previously used to successfully probe associations with color and music [25]. Participants were allowed to control their sniffing within each trial, and there was a minimum of a 20 second pause between each pen.

### Analyses

The color responses of each participant for each odor can be found online at http://figshare.com/authors/Carmel_Levitan/559155. For each odor, one pattern of choices was compiled, pooling choices from all participants of one population sample. A color pattern consists of 72 features; two for each of the 36 colors participants could choose; one for the number of congruent and one for the number of incongruent choices. Separate color patterns were made from each of the population samples. For each odor within each culture, we calculated the chi-square associated with the pattern of congruent and incongruent color choices.

A pattern classification analysis served to assess the stability of color-odor associations within each culture. The Fisher's Linear Discriminant classifier (for a comparison of classifiers see e.g. [26]) was trained on two-thirds of the data and subsequently classified the remaining one-third of the data, by identifying the odor for each color-pattern. Leave-one-sample-out cross-validation ensured that all parts of the data were equally contributed to test and training data. The procedure was then repeated after shuffling the cross-validation folding 1,000 times, to obtain a stable accuracy sample. The same cross-validation and resampling procedures were applied, while permuting the colors for each of 10,000 repetitions, to obtain a distribution of accuracy values under the null hypothesis that color-odor associations are not stable. The accuracy sample for each culture was then compared to the null-distribution to determine its statistical significance.

To quantify the similarity structure, or isomorphism, of odor-color associations within a culture, we created one representational dissimilarity matrix (RDM; [22]) for each population. The RDM has as many rows and columns as there are odors, and each cell in the matrix stands for the dissimilarity between the color-pattern of the odors in the respective row and column. The diagonal of the matrix therefore contains only the value of 0 for perfect similarity of an odor's color-pattern with itself. The dissimilarity was defined as 1 - correlation between the two patterns (Pearson's r). Using bootstrapping (by repeatedly shuffling the colors and resampling the RDM), a null-distribution was created which displays the variance in dissimilarity.

To quantify the difference between cultures, the second-order isomorphism was calculated as the pair-wise dissimilarity between the culture-specific RDMs, compiled as one inter-culture RDM.

## Results and Discussion

**Figure 2:**
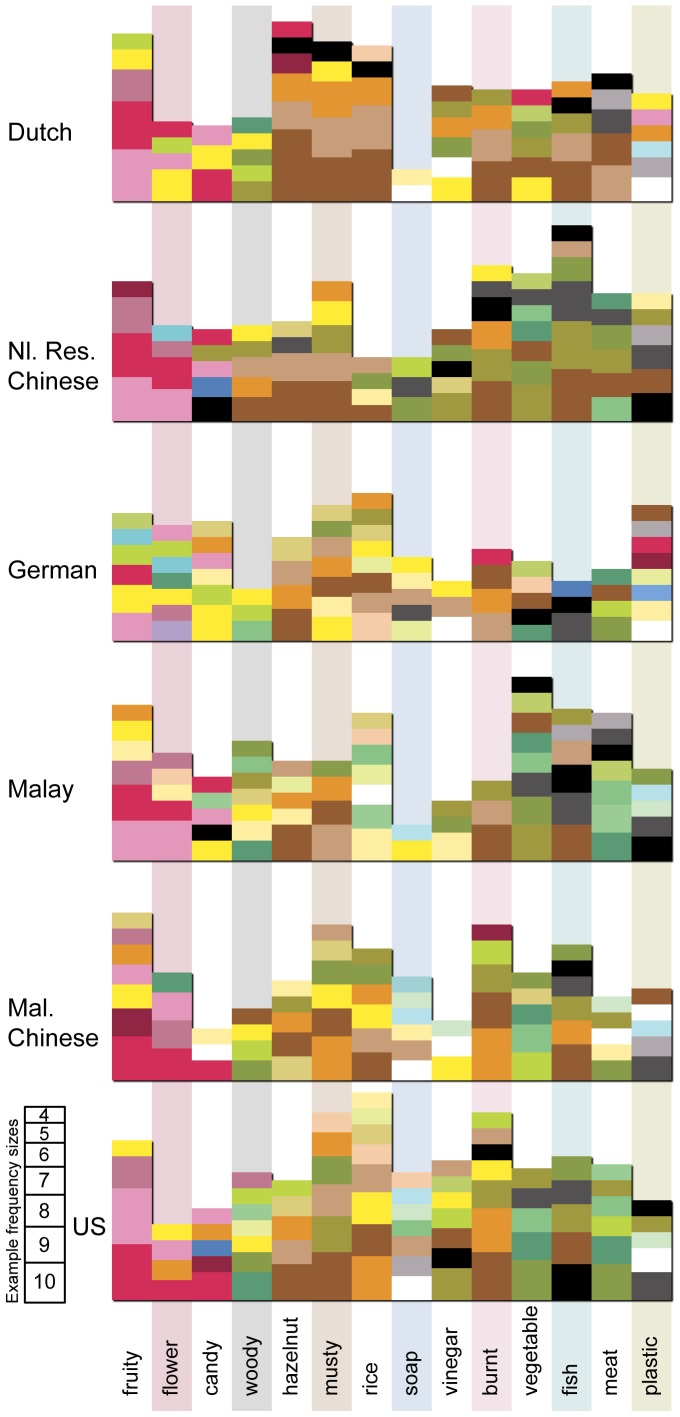
Color congruency for each odor in each culture. Colors per odorant per country are ordered by frequency (most frequent are shown lowest on their respective y-axis). Frequency is represented by the height of each color box; the box on the right of the figure shows the height a given box must be for there to be 10, 9, 8 etc. ratings of that color for a given odorant. Boxes have been given a slight shadow to help with the perception of harder to see light colors. The background bars are only colored so as to help with reading the figure.

Figure 2 depicts the most frequently selected congruent color matches for each odor within each culture. For instance, the fruity odor tended to be associated with pink and red colors, while the musty odor was more associated with browns and oranges. All of our data analyses used both congruent and incongruent choices to fully map out the color-odor association space, and for each color-odor combination, congruent and incongruent selections were counted separately (allowing us to see differences between colors that were rarely or never selected and colors that were selected as both congruent and incongruent by different participants for the same odor). All of the 14 chi-square values within each of our six groups were statistically significant. Chi-square values, listed in Table 1, ranged from 35.6–41.2, *p* all below 0.0002. A Bonferroni-corrected cutoff value for significance for the 84 comparisons would result in a threshold for significance of 0.0006. This confirms that, within each group, there were consistent patterns of color choices (including both congruent and incongruent) for each odor. This result is consistent with those of other studies that only had participants make congruent matches (e.g., [2]–[5]).

**Table 1:** Chi-square and p-values comparing the pattern of color choices for each odor in each culture to that which would be expected if color choices were random.

	Dutch		Netherlands-residing Chinese		German		Malay		Malaysian-Chinese		US	
Odor	*χ^2^*	*p*	*χ^2^*	*p*	*χ^2^*	*p*	*χ^2^*	*p*	*χ^2^*	*p*	*χ^2^*	*p*
fruity	39.77	.000039	39.89	.000037	41.2	.000022	38.69	.00006	37.12	.00011	37.12	.00011
flower	40.57	.000029	39.71	.00004	39.45	.000044	38.68	.00006	35.81	.000182	35.81	.000182
candy	38.68	.00006	40.61	.000028	38.95	.000054	39.2	.000049	36.07	.000165	36.07	.000165
woody	39.3	.000047	40	.000036	38.36	.000068	38.47	.000065	35.67	.000192	35.67	.000192
hazelnut	39.85	.000038	39.82	.000038	38.66	.000061	38.6	.000062	35.71	.000189	35.71	.000189
musty	40.51	.000029	39.01	.000053	39.5	.000044	38.37	.000068	37.23	.000105	37.23	.000105
rice	40.65	.000028	39.12	.000051	39.43	.000045	37.92	.000081	37.41	.000099	37.41	.000099
soap	39.36	.000046	38.82	.000057	39.11	.000051	38.12	.000075	35.62	.000196	35.62	.000196
vinegar	38.7	.00006	39.11	.000051	38.22	.000072	38.5	.000064	35.63	.000195	35.63	.000195
burnt	39.77	.000039	38.6	.000062	39.29	.000047	38.27	.000071	35.67	.000192	35.67	.000192
vegetable	39.59	.000042	38.35	.000068	37.93	.000081	38.24	.000071	36.66	.000132	36.66	.000132
fish	38.92	.000055	38.56	.000063	39.32	.000047	41.08	.000023	35.67	.000192	35.67	.000192
meat	38.68	.00006	38.56	.000063	38.05	.000077	38.33	.000069	36.01	.000169	36.01	.000169
plastic	38.67	.00006	40.08	.000035	38.72	.000059	38.66	.000061	36.32	.00015	36.32	.00015

These chi-square tests confirmed the consistency of each of the odor's color associations separately. The pattern classifier evaluates the degree to which this consistency, and the specificity of odors' color patterns among each other, is enough to identify the odors solely on the basis of their color associations. If there were no consistency between participants' color-odor associations within a culture, the pattern classifier would be expected to identify one odor out of fourteen correctly just by chance, and thus have an accuracy of 7%. Classifiers for four out of six cultures performed significantly better than chance; US accuracy was 22% (SD = .042, *p*<.01), German accuracy was 19% (SD = .046, *p*<.01), Malaysian Chinese accuracy was 16% (SD = .043, *p* = .02), and Dutch accuracy was 15% (SD = .035, *p* = .03). Performance for the other two cultures was not significantly higher than chance; Malay accuracy was 9% (SD = .031, *p* = .26) and the Netherlands-resident Chinese accuracy was also 9% (SD = .031, *p* = .33).

**Figure 3:**
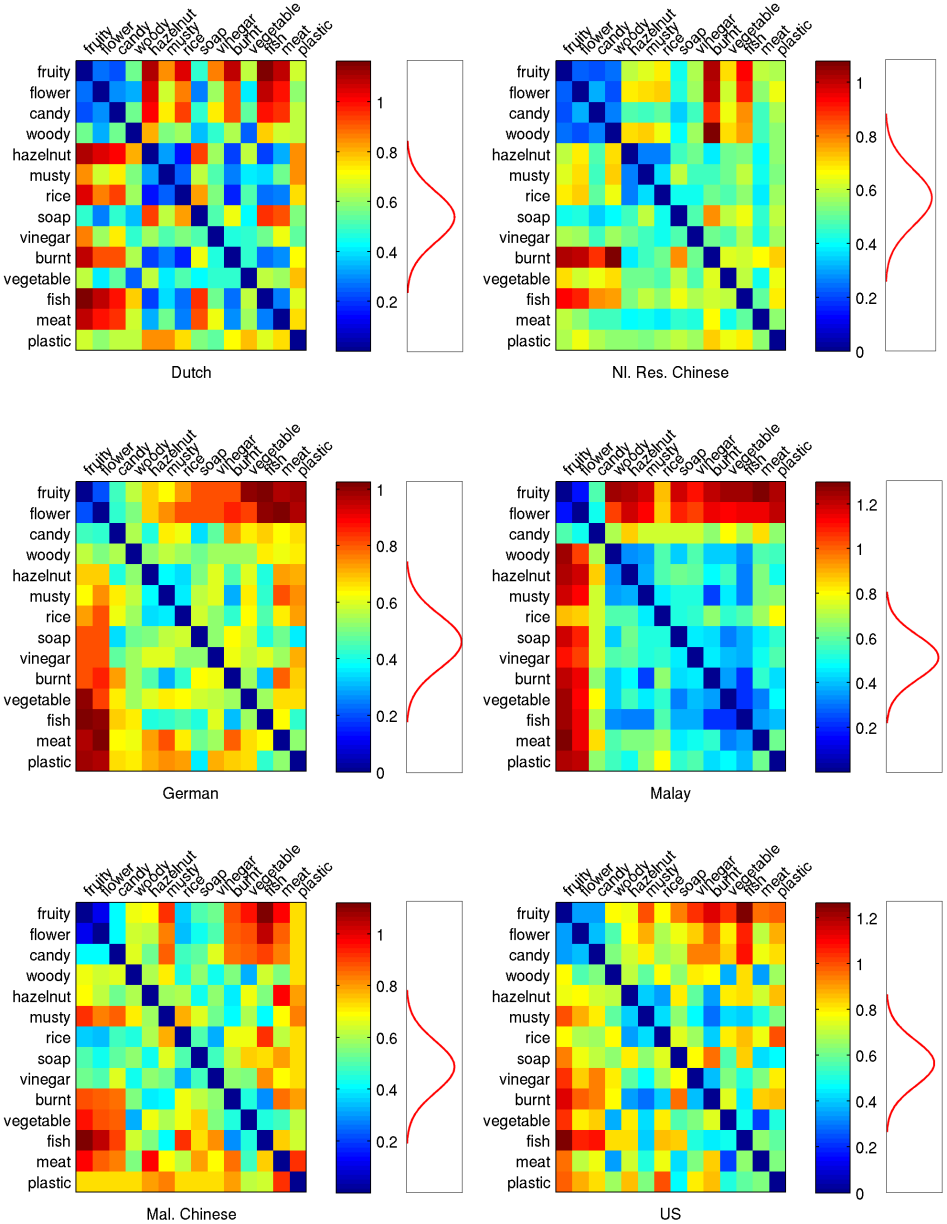
Odor representation by culture. One Representational Dissimilarity Matrix (RDM) for each of the six populations sampled. Both axes of each matrix represent the 14 odors. Each cell in the matrix indicates the degree of dissimilarity between the color-patterns of the respective odors in that row and column. Warmer colors indicate higher dissimilarity. The dark blue diagonal indicates the perfect similarity of the odors with themselves. The representational geometry, or the spatial configuration of clusters of high and low dissimilarities, shows differences and commonalities in each culture. Along the color legend, a line graph shows a bootstrapped null distribution of pattern dissimilarities.

Because we wanted to better understand the patterns of color choices (congruent and incongruent) for each odor, we calculated the RDMs within each culture, shown in Figure 3. This allows us to take advantage of potential similarities between colors and odors without presupposing a particular representation of those similarities. Within the US, for instance, clusters of odors that had similar patterns of color choices were fruity, flower, and candy; hazelnut, musty, burnt, vinegar, and rice; and meat, woody, and vegetable. We also calculated RDMs using only the congruent choices, and found that they were not as successful in finding similarities in patterns of color-odor choices; while this may be in part due to a reduction in data and thus in statistical power, we believe that the incongruent choices are important for uncovering the underlying color-odor space. By having each participant generate six responses (three congruent and three incongruent) to each odor, we could thoroughly map the color-odor association space; in cases where multiple colors are likely to be associated to a single object, allowing participants to select more than one color might better capture the difference between, say, cherries and strawberries, which might both be associated most strongly with red but have different secondary associations. Our participants did not report any difficulty with our procedure, and we note that Palmer et al. [25] had participants match five congruent and five incongruent colors to pieces of music. Because our study did not ask participants to rank their choices, we cannot test whether having our participants generate multiple responses provided more useful information than a single response would have, but future research could directly assess this.

**Figure 4:**
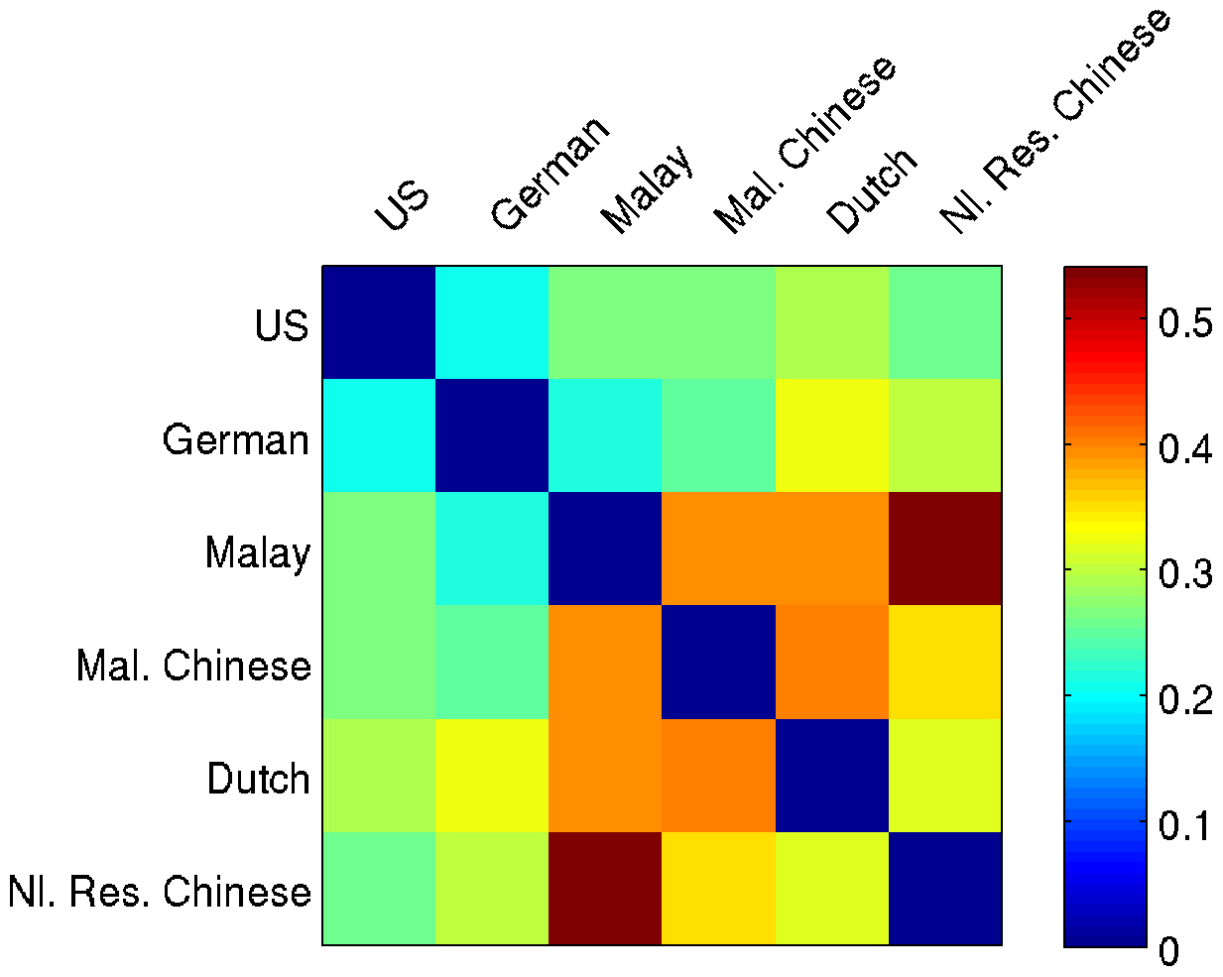
Crosscultural dissimilarity of representation. Representational Dissimilarity Matrix comparing cultures. Both axes represent the six cultures. Each cell in the matrix indicates the degree of dissimilarity between the respective cultures' odor representation geometry (Figure 3). The dark blue diagonal indicates the perfect similarity of the cultures with themselves.

We computed the cross-culture RDM shown in Figure 4 by comparing the overall color-odor associations for each color. We had initially expected that, if some differences between cultures were to emerge, geographical and cultural similarities would lead to similar patterns as in Pangborn et al.'s 1998 study [15]; thus we had predicted that German and Dutch choices would be quite similar and that Malaysian-Chinese choices would be similar to those of both Malay and Netherlands-residing Chinese. None of those predictions about the degree of correspondence between cultures, however, were supported by the data. The most similar color-odor associations were between the US and Germany and between German and Malay participants. The largest differences were between Malay and Netherlands-resident Chinese, and between Dutch and Malaysian-Chinese, and the Malay also differed notably from the Dutch and the Malaysian-Chinese. Overall, the US participants showed the most similarities to other cultures while the Malay participants were the most different from participants in other cultures. These differences could be due to patterns in dietary habits, the role of fragrance in each society, or other social factors. Moreover, our study cannot isolate culture from other factors that may have caused our samples to differ by group. Past research has shown that female and male participants do not differ in their color-odor associations [5], but other actual or potential differences between our groups, such as age, travel experience, and frequency of cooking, could contribute to the differences we observed in color-odor associations.

The cross-cultural results indicate that color-odor associations – while fairly consistent within a culture – differ across cultures. This pattern argues against the notion that color-odor associations are structural, as structural correspondences would be largely universal. Instead, the results favor statistical or semantically-mediated learning of color-odor correlations. This contrasts with the recent claim that crossmodal correspondences between odors, musical notes, and geometrical shapes are likely to be structural in nature [27]. Spence and Deroy [28] point out that different pairings of modalities may have different types of crossmodal correspondences, which in turn could influence the automaticity of those correspondences. Our result underscores the importance of this theoretical point.

It has been shown previously that odor perception can be greatly influenced by language or verbal labels [29]. While it is possible that our results are due to semantic differences, we attempted to minimize effects of language by selecting odors that were relatively general (e.g., fruity rather than strawberry, vegetable rather than broccoli) and we created an interface and procedure that was relatively non-verbal. Moreover, people's ability to label odors is “astonishingly bad” [30] (see also [31]), though this itself may be culturally specific [32]. Further research could tease out the effect of language by asking some participants to identify the odor before making color choices and exploring how color choices differ as a result of identification, though this would necessitate a very large number of participants.

Associative learning has been shown to influence odor perception; for instance, odors that have been paired together are later judged to be more similar [33] and odors that have been paired with sucrose are later judged to smell sweeter [34]. Odors can even become associated with emotions, which in turn shape behavior [35]. Thus it is plausible that color's many effects on odor [6] also arise via associative learning. The present study does not allow us to assess whether cultural differences in color-odor associations are due to direct associations between colors and odors or due to cultural differences in perceived intensity and pleasantness which in turn influence color-odor matches; it is likely that associative learning influences all of these parameters. However, it could be that some features, such as intensity, are structurally mapped while others are not (see [27]).

In conclusion, we have shown that that there are substantial cross-cultural variations in color-odor associations. This is consistent with the notion that these associations are learned through experience.
